# *Alnus Sibirica* Extracts Suppress the Expression of Inflammatory Cytokines Induced by Lipopolysaccharides, Tumor Necrosis Factor-α, and Interferon-γ in Human Dermal Fibroblasts

**DOI:** 10.3390/molecules24162883

**Published:** 2019-08-08

**Authors:** Jeongyoon Choi, Sunghee Moon, Hyemi Bae, Young-Won Kim, Donghee Lee, Seongtae Kim, Yelim Seo, Hye Soo Wang, Young Wook Choi, Min Won Lee, Jae-Hong Ko, Inja Lim, Hyoweon Bang

**Affiliations:** 1 Department of Physiology, College of Medicine, Chung-Ang University, Seoul 06973, Korea; 2 Laboratory of Pharmacognosy and Natural Product based Medicine, College of Pharmacy, Chung-Ang University, Seoul 06973, Korea; 3 College of Pharmacy, Chung-Ang University, Seoul 06973, Korea

**Keywords:** skin inflammation, atopic-like dermatitis, inflammatory stimulators, cytokines, *Alnus sibirica*, anti-inflammatory

## Abstract

The effects of *Alnus sibirica* (AS) extracts on cytokine expression induced by inflammatory stimulants were examined in human dermal fibroblasts (HDFs) and RAW264.7 cells. The anti-oxidative effect and effect on cell viability of AS extracts were evaluated, and four extracts with the highest anti-oxidative effects were selected. HDFs and RAW264.7 cells were treated with inflammatory stimulants, and the expression of cytokines involved in acute (IL-6 and IL-10) and chronic (IL-18) inflammation, the initiation of the immune response (IL-33), and non-specific immune responses (IL-1β, IL-8, and TNF-α) were determined using a reverse-transcription polymerase chain reaction. LPS increased the expression of all the cytokines, except for IL-18; however, AS extracts, particularly AS2 and AS4, reduced this increase, and TNF-α treatment markedly increased the expression of cytokines related to non-specific immune responses. IFN-γ treatment induced no significant changes, except for increased IL-33 expression in HDFs. AS extracts inhibited the increase in the expression of IL-33 and other cytokines in HDFs. Thus, the exposure of HDFs and RAW264.7 cells to inflammatory stimulants increased the expression of cytokines related to all the inflammatory processes. HDFs are involved not only in simple tissue regeneration but also in inflammatory reactions in the skin. AS2 and AS4 may offer effective therapy for related conditions.

## 1. Introduction

Inflammation, the response to stimuli such as infections or noxious substances that induce tissue damage, involves many reactions. Cytokines, which are secreted as part of the immune response, are important factors that determine the extent of inflammation. Interleukin (IL)-1β, IL-6, IL-8, IL-10, IL-18, and tumor necrosis factor (TNF)-α are the essential cytokine mediators that connect immune cells and inflammation. Persistent inflammation caused by the excessive production of active cellular substances and pro-inflammatory mediators leads to various types of inflammation [1]. In addition, it causes many diseases, including skin inflammation [2], sepsis [3], asthma [4], pulmonary fibrosis [5,6], rheumatoid arthritis [7], and atherosclerosis [8]. Therefore, an understanding of the relationship between cytokines and inflammation is essential for the appropriate treatment of inflammatory diseases [2,9,10].

On the basis of preclinical and clinical studies, the cytokines can be subdivided in at least 5 subfamilies as follow: The pro-inflammatory type 1 helper T (Th1)/Th17 cytokines, the anti-inflammatory Th2/Th3 cytokines and the Th9 cytokine represented by IL-9. Th1 and Th17 cytokines primarily mediate pro-inflammatory effects. They are produced by Th1 and Th17 cells and M1 macrophages and include, among others, IL-1, TNF, interferon gamma (IFN-γ), IL-12, IL-18, IL-22 IL-23, IL-17. These cytokines are thought to contribute to the thought the induction and maintenance of cell-mediated autoimmune diseases such as rheumatoid arthritis, type 1 diabetes, arthritis, multiple sclerosis and Guillain barre’ syndrome [11,12].

The anti-inflammatory cytokines such as IL-4, IL-10, IL-13, IL-35 and transforming growth factor-beta (TGF-β) are primarily produced by M2 macrophages, Th2 and Th3 cells. They have been shown to decrease inflammation and are implicated in immunoglobulin E (IgE) mediated allergic disorders [13,14,15]. Certain diseases such as systemic lupus erythematosus are characterized by the simultaneous contribution of both Th1 and Th2 cytokines [16]. The role of IL-9 of the immune responses is less defined but it is receiving increased attention as another cytokine with important potential implications in the pathogenesis of autoimmune diseases [17,18,19,20].

Recently, the incidence of immune-mediated skin disease has increased owing to environmental pollution, the westernization of lifestyle and diet, and stress. The multiple causes of atopic dermatitis (AD) include genetic and environmental factors, imbalanced immune function, and abnormal skin barrier function [21,22,23]. Patients with AD display skin symptoms such as dryness, itching, and roughness [24]. Among these, itching is the primary clinical symptom of AD. Lymphocytes, macrophages, and granular mast cells can infiltrate the skin lesions of AD. AD is divided into acute and chronic types, depending on the related immune response. Acute AD accounts for approximately 70–80% of cases, and is characterized by the elevation of serum IgE levels and Th2 cytokines (e.g., IL-4, IL-6, and IL-13) [25]. In chronic AD, Th1 cytokines (e.g., IL-12 and IFN-γ) play more prominent and important roles. AD has a high rate of recurrence, and most cases progress to the chronic state. Moreover, effective treatments have not yet been established.

Fibroblasts are actively involved in the immune response of the skin and influence the transition from the acute to the chronic phase of AD [26]. They also have a role in the provision of structural support and can secrete and respond to cytokines, chemokines, and growth factors (GFs). Fibroblasts also maintain the homeostasis of adjacent cells and are essential for tissue development, differentiation, remodeling, and repair; in addition, they coordinate inflammatory infiltration [27].

Macrophages are strategically located throughout the body and perform a critical immune surveillance function, continuously monitoring their immediate surroundings for signs of tissue damage or invading organisms. When stimulated by poisoned lymphocytes, they induce other immune cells to respond to phagocytosed danger signals and/or are detected by cell surface receptors [28]. During inflammation, macrophages are activated, respond to a variety of infectious agents, and produce inflammatory mediators such as TNF, ILs, and leukotrienes [29].

Many cytokines are involved in skin inflammation. For example, IL-1β acts as a chemoattractant that stimulates T cells, promotes B cell maturation, and increases the activity of natural killer (NK) cells [30,31]. Monocytes, macrophages, B cells, dendritic cells (DCs), endothelial cells, and neutrophils secrete IL-1β [32,33,34]. IL-6 promotes the final differentiation of B cells into plasma cells, the stimulation of antibody secretion, and the induction of the synthesis of acute inflammation-inducing factors. Monocytes and macrophages mainly secrete IL-6 [35]. IL-8 increases neutrophil activation and promotes the exocytosis of damaged lysosomal enzymes and the production of lipid mediators [36,37]. It is involved in the induction of nuclear factor-kappa B (NF-kB) receptor activation in fibroblasts [38]. IL-10 has many roles in immunoregulation and inflammation: it inhibits the secretion of costimulatory molecules in macrophages and activates the secretion of Th2 cytokines when inflammation develops [39]. IL-18 acts on Th1 cells and NK cells, and induces the production of inflammatory cytokines (e.g., IFN-γ) [40,41]. IL-33, which acts as an alarmin for the initiation of inflammation, is produced in endothelial/epithelial cells, hematopoietic cells (especially macrophages), and a wide variety of immune cells (e.g., Th2 cells, CD8+ T cells [42], eosinophils [43], basophils [44], NK cells [45], DCs [46], and mast cells [31,47]). The disruption of the epidermal barrier induces the excessive production of IL-33, which initiates skin inflammation and leads to the dysregulation of immunomodulatory proteins [48,49]. TNF-α is involved in fever, the activation of granulocytes and T cells, and the induction of sepsis [50]. In fibroblasts, TNF-α leads to the excessive production of inflammatory cytokines through the NF-kB pathway [51]. TNF-α also delivers inflammatory signals from monocytes and macrophages to other immune cells and causes dermatitis, asthma, and allergic inflammation [29].

**Figure 1:**
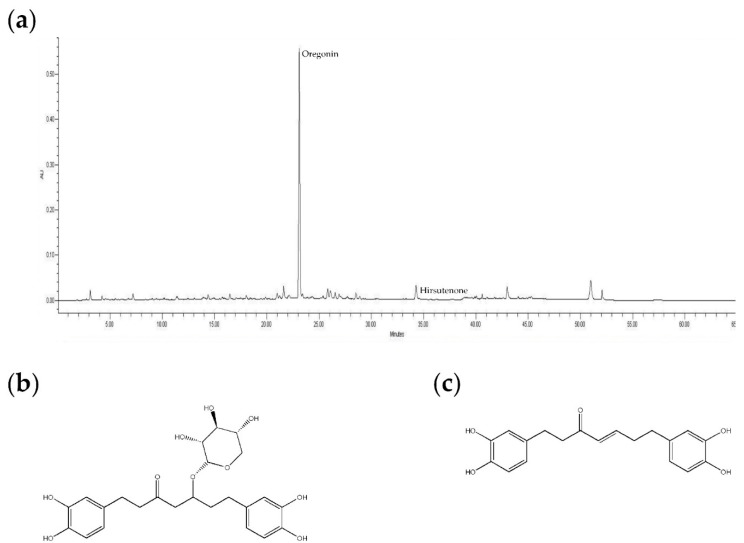
HPLC pattern of Alnus sibirica (AS) and major compounds in AS extracts: (**a**) HPLC pattern of AS; (**b**) The chemical structure of oregonin; (**c**) The chemical structure of hirsutenone (1,7-bis-(3,4-dihydroxyphenyl)-4-heptene-2-one).

*Alnus sibirica* (*A. sibirica*; AS) is a deciduous tree of the family Betulaceae and the genus *Alnus*. More than 15 species are known to be native to Korea; they are also found throughout North Korea, China, Russia, and Japan [52]. The species *A. japonica*, *A. firm*, *A. maximowiczii*, and *A. hirsute var. sibirica* are native to Korea. In oriental medicine, AS bark is called saegjeog-yang, and has been used to treat the heat, hemorrhage, diarrhea, gastrointestinal disorders, lymphatic diseases, and cancer [53,54]. AS contains phenolic compounds such as flavonoids [55], tannins, and triterpenoids [56,57,58,59,60], including a series of diarylheptanoid triterpenoids. These compounds are reported to have antioxidant, anti-inflammatory, and anti-AD activities [61,62]. Oregonin (Figure 1a,b) and hirsutenone (Figure 1a,c) are the main components of diarylheptanoids in AS extracts. It has been reported that they have antioxidant, anti-inflammatory [58], anti-atopic dermatitis [63], and cytotoxic [64] properties. Lee et al. [65] reported that the oral administration of fermented AS extracts (FAS) reduced the expression of IgE, IL-4, and IFN-γ in splenocytes from BALB/c mice with atopic-like dermatitis. However, they reported only the mitigating effects on systemic inflammation and did not describe the effects of AS extracts on the various cytokines associated with skin inflammation.

The effect of AS extracts on the cytokines involved in skin inflammation is not well known. In this study, we used two types of cells: fibroblasts, which may have a direct effect on skin inflammation, and macrophages, which are the primary type of cells related to immune-mediated inflammation. We observed the expression patterns of various cytokines (IL-1β, IL-6, IL-8, IL-10, IL-18, IL-33, and TNF-α) in these cells after treatment with inflammatory stimulants (Lipopolysaccharide (LPS), TNF-α, and IFN-γ), and identified the major cytokines involved. Further, we explored the effects of AS extracts on the expression patterns of these cytokines.

## 2. Results

### 2.1. Measurement of 2,2-Diphenyl-1-Picrylhydrazyl (DPPH) Radical Scavenging Activity

**Figure 2:**
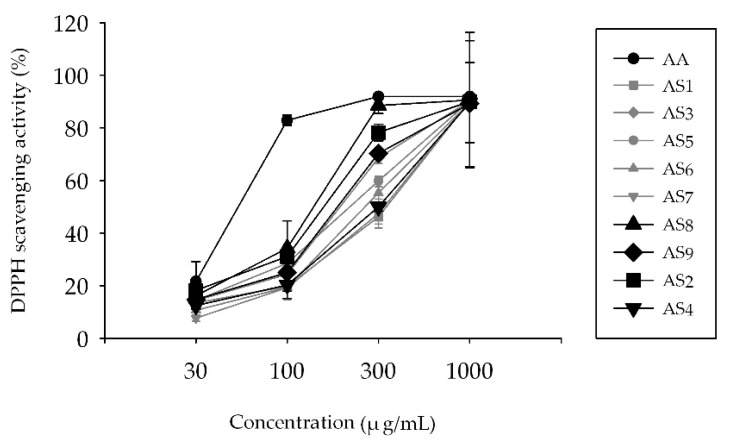
DPPH radical scavenging activity of AS extracts (AS1–AS9). X-axis: concentration of applied compound, Y-axis: DPPH radical scavenging activity (%). The results are presented as the mean ± S.D. of experiments (*n* = 6).

The antioxidant capacity of AS extracts isolated from nine extracts of AS (AS1–AS9) was examined by using the DPPH radical scavenging assay (Figure 2). At 30 μg/mL, the antioxidant capacity of ascorbic acid (AA) and all AS extracts was approximately 21% and 20% or below, respectively. At 100 μg/mL, the antioxidant capacity of AA was 80%; the capacities of AS2 and AS8 were 30%; the capacities of AS3, AS5, and AS9 were approximately 25%; and the capacities of AS1, AS4, AS6, and AS7 were 20% or below. At 300 μg/mL, the effects of the AS extracts were varied. AS8 had the highest antioxidant capacity (88%); the capacities of AS2, AS3, and AS9 were approximately 70%; and the capacities of AS1, AS4, AS6, and AS7 were 50%. At 1000 μg/mL, the antioxidant capacity of all the AS extracts was approximately 90%. We selected AS2, AS4, AS8, and AS9 for the 3-(4,5-dimethylthiazol-2-yl)-2,5-diphenyltetrazolium bromide (MTT) assay and reverse-transcriptase polymer chain reaction (rtPCR) analysis after the consideration of several factors, including the antioxidative effect of the AS extract, the extraction efficiency, and the availability of the raw material.

### 2.2. Measurement of Cell Viability

**Figure 3:**
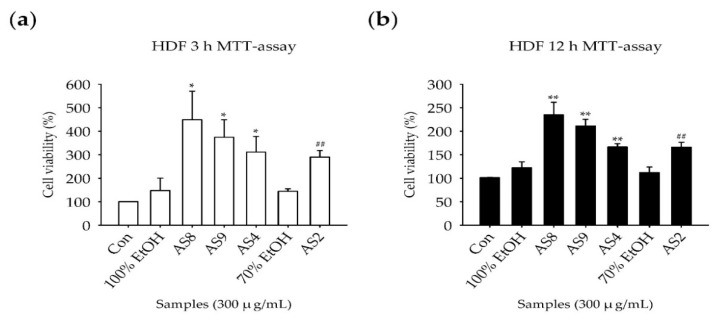

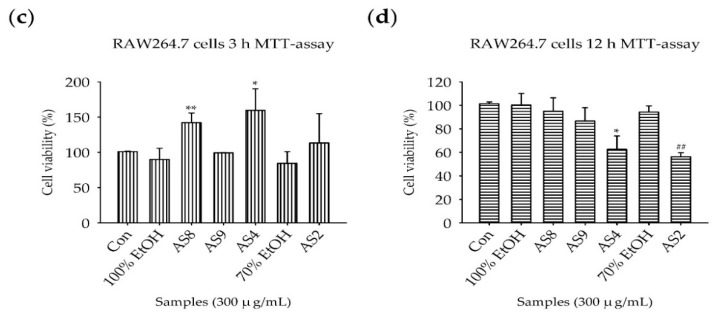
Cytotoxicity of AS extracts to human dermal fibroblasts (HDFs) and RAW264.7 cells: AS extract treatment for (**a**) 3 h in HDFs; (**b**) 12 h in HDFs; (**c**) 3 h in RAW264.7 cells; and (**d**) 12 h in RAW264.7 cells. Each extract was tested at 300 μg/mL or 1000 μg/mL for 3 h or 12 h before the 3-(4,5-dimethylthiazol-2-yl)-2,5-diphenyltetrazolium bromide (MTT) assay was performed. X-axis: Sample treatment and dose, Y-axis: cell viability (%). The results are presented as the mean ± S.D. of triplicate experiments (*n* = 3). Statistical significance was assessed by *t*-tests represented as follows: * *p* < 0.05 and ** *p* < 0.01 vs. 100% Ethanol; ## *p* < 0.01 vs. 70% Ethanol; the result of cell proliferation, not cell death, except for the 12-h results of RAW 264.7 cells.

The MTT assay was used to examine the extent of cell death induced by treatment with AS2, AS4, AS8, and AS9 in HDFs and RAW264.7 mouse macrophages. As the experimental protocols for stimulation with LPS require incubation for 3 h and 12 h with TNF-α and IFN-γ, respectively, we evaluated the effect of AS extracts on cell death after 3 h and 12 h. Vehicle control was examined to determine the effect of 70% and 100% ethanol on the samples. Treatment with vehicle and all the AS extracts for 3 h and 12 h did not lead to cell death (Figure 3), except for the treatment of AS2 and AS4 for 12 h in RAW264.7 cells (Figure 3d). Most AS extracts enhanced cell proliferation rather than induced cell death but to different extents. In HDFs, cell proliferation was approximately threefold higher than the control after treatment for 3 h (Figure 3a), and approximately twofold higher after treatment for 12 h (Figure 3b). In RAW264.7 cells, the induction of proliferation was not large, with the growth approximately 1.5-fold that of the control after treatment with AS8 and AS4 for 3 h. The precise mechanism of the proliferative effect will be analyzed in future experiments.

### 2.3. Reverse-Transcription Polymerase Chain Reaction

The synthesized cDNA was analyzed by PCR. Changes in the expression of cytokines and the effects of AS extracts on these changes were evaluated from the area and brightness of the identified band. ImageJ software (National Institute of Mental Health, Bethesda, MD, USA) was used for analysis. Each band was normalized to the expression of glyceraldehyde 3-phosphate dehydrogenase (GAPDH) and compared with the control band.

### 2.4. Effects of LPS

**Figure 4:**
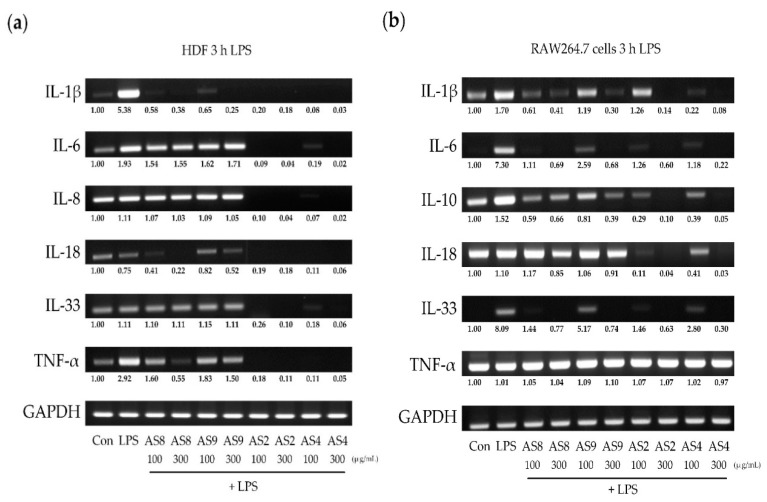
Changes in cytokine expression induced by LPS in (**a**) human dermal fibroblasts (HDFs) and (**b**) RAW264.7 mouse macrophages treated with AS extracts.

In HDFs, LPS treatment resulted in a fivefold increase in IL-1β expression (Figure 4a) and a twofold increase in TNF-α. All the AS extracts significantly decreased IL-1β and TNF-α; in particular, AS2 and AS4 almost completely inhibited all the cytokines. In RAW264.7 cells treated with LPS, IL-1β and IL-10 were upregulated by up to 1.5-fold, and IL-6 and IL-33 were upregulated by more than sevenfold (Figure 4b). AS8 and AS9 treatments significantly reduced the expression of cytokines induced by LPS. Similar to HDFs, treatment with AS2 and AS4 almost completely suppressed the expression of all cytokines except for TNF-α (threefold suppression of IL-1β and IL-10, and 6-fold suppression of IL-6 and IL-33).

### 2.5. Effects of TNF-α

**Figure 5:**
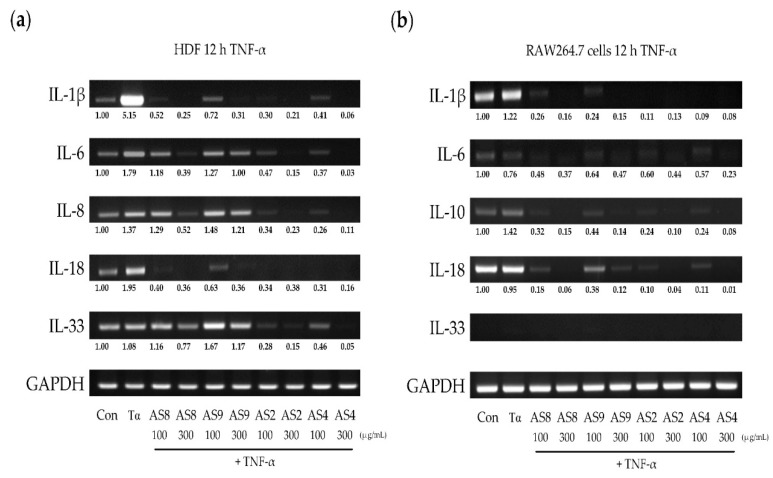
Changes in cytokine expression induced by tumor necrosis factor (TNF-α) after treatment of (**a**) HDFs and (**b**) RAW 264.7 cells with AS extracts.

When HDFs were treated with TNF-α, different upregulation of the cytokines was observed (Figure 5a). IL-1β, which is closely related to inflammation, was increased by more than fivefold and inhibited more than 10-fold by treatment with all AS extracts. IL-6 and IL-8 were increased by approximately 1.5-fold. It was not changed by the treatment of AS9, but was decreased more than threefold by the treatment of AS2, AS4, and AS8. The increase in IL-18 expression was inhibited more than threefold by treatment with all the AS extracts. IL-33 was not significantly changed after treatment with TNF-α but was 0.3-fold lower than the control after treatment with 300 μg/mL AS8, and more than twofold lower after treatment with AS2 and AS4. In RAW264.7 cells, IL-1β was increased by 1.2-fold, and IL-10 was increased by 1.4-fold (Figure 5b). All the AS extract treatments significantly inhibited the expression of all cytokines, except for IL-33.

### 2.6. Effects of IFN-γ

**Figure 6:**
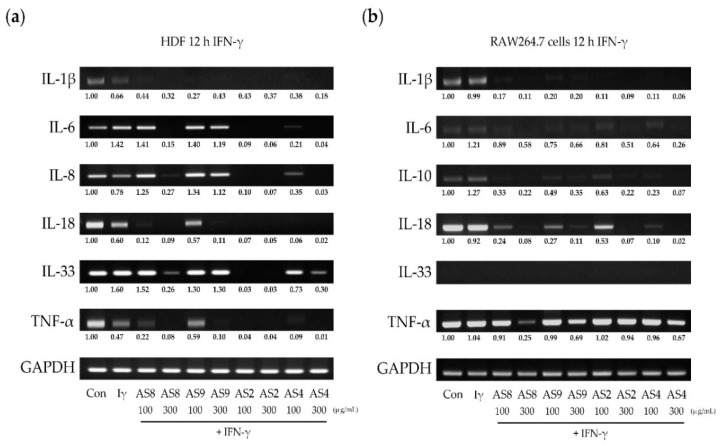
Changes in cytokine expression induced by IFN-γ in (**a**) HDFs and (**b**) RAW 264.7 cells treated with AS extracts.

When HDFs were treated with IFN-γ, IL-6 and IL-33 were upregulated by 1.4-fold and 1.9-fold, respectively (Figure 6a). All the AS extracts inhibited the IFN-γ-induced expression of IL-1β. Treatment with AS2, AS4, and AS8, but not AS9, completely inhibited the expression IL-6, IL-8, and IL-18. Treatment with 300 μg/mL of AS2, AS4, and AS9 strongly inhibited IL-33. For TNF-α, the reduced expression was further reduced by treatment with AS extracts, except for 100 μg/mL of AS9. In RAW264.7 cells, IL-6 and IL-10 were slightly increased, by 1.2-fold. All the cytokines, except for IL-33 and TNF-α, which were not expressed, were strongly inhibited by all the AS extracts. The expression of TNF-α was inhibited more than twofold by treatment with AS2, AS8, and AS9.

## 3. Discussion

Oregonin and hirsutenone are the main components of diarlyheptanoids in AS extracts used in our experiments. We were expected that they played an important role in the effects of the ASs used in this study. However, in the case of the whole extract, various other ingredients may also be effective. Therefore, it is important to observe the effect of the whole extract, although the effect of a single substance is also important. S. E. Choi et al. [59] reported that *Alnus japonica* (AJ) extracts containing oregonin and hirsutenone as major components exhibited anti-atopic dermatitis effects in NC/Nga mice induced with atopic-like dermatitis. Since the orgonin and hirsutenone are known to be the major components in the AS used in this study, substantial parts of the effect of this study were also attributed to these two substances. Antioxidant effects are closely related to anti-inflammatory effects. When inflammatory reactions occur in the body, a series of reactive oxygen species (ROS), such as nitrogen monoxide (NO), superoxide anion (O_2_^−^), and hydrogen peroxide (H_2_O_2_) are produced in abundance. This increases oxidative stress, which results in further stimulation of the inflammatory response. The antioxidant activity of extracts from AS leaves and bark was observed by using the DPPH radical scavenging assay. From 300 μg/mL, the DPPH activity of all the AS extracts increased dramatically. AS8 (extract of barks from Jacheon), AS9 (leaves from Jacheon), and AS2 (leaves from Hamyang) showed the strongest antioxidant effect. We added AS4 (barks from Hamyang) for comparison with AS2. All the subsequent experiments were performed with these four selected AS extracts.

The effect of each of these AS extracts on cell death was evaluated by using the MTT assay. In HDFs, treatment with all the AS extracts for 3 h and 12 h did not result in cell death, but instead resulted in an increase in cell proliferation. However, in RAW264.7 cells, approximately 40% viability was observed after treatment with 300 μg/mL of AS2 and AS4 for 12 h. At 100 μg/mL, cell death did not occur. High concentrations of AS2 and AS4 for 12 h may result in direct toxicity to RAW 264.7 cells, so the effect of cell death should be considered in experiments measuring cytokine expression. To exclude the effect of cell death in this study, the concentration of RNA was measured, and an equal amount was used for cDNA synthesis. The rtPCR results were normalized with the density of GAPDH in the control group.

After the treatment of HDFs and RAW264.7 cells with inflammatory stimulants, the expression of inflammatory cytokines was examined, and the effects of AS extracts were analyzed. AD has an acute phase (IgE-derived Th2 immune response) and a chronic phase (cell-mediated Th1 immune response) [66]. Th2 cytokines, such as IL-4, IL-5, IL-6, IL-10, IL-13, IL-25, and IL-31, are involved in the acute phase of AD; Th1 cytokines, such as IL-2, IL-12, IL-18, and IFN-γ, are involved in the chronic inflammatory stage. The negative feedback loop of Th1 and Th2 cytokines are known to be an important mechanism that causes persistent skin inflammation in AD. An increase in the expression of Th2 cytokines inhibits Th1 cytokines, and an increase in Th1 cytokines suppresses Th2 secretion. IL-4 and IL-13, which are directly related to the development of AD, were not observed in the HDFs used in this study. IL-6 is an essential cytokine of the acute phase immune responses owing to its transmission of the signal to defend against pathogen attack and stimulate host defenses in response to tissue damage [67,68]. IL-10 is an essential immunomodulatory cytokine that is produced in Th2 cells, but it is also produced in B cells, mast cells, and macrophages [69,70,71,72]. In many inflammatory disease models, such as chronic enterocolitis [73,74], skin inflammation [75], endotoxin shock [76], and encephalomyelitis [77], IL-10 plays a central role in the inflammatory response. In this study, IL-6 and IL-10 were investigated as the cytokines involved in the acute inflammatory response. IL-12 and IFN-γ were mainly involved in the chronic phase of AD, but IL-12 was not highly expressed in HDFs; therefore, IFN-γ was used as an inflammatory stimulant in our experiments. IL-18 is known to be a useful index of chronic inflammation in keratinocytes (Kc) [78] and macrophages [79], and is strongly expressed in HDFs and was selected to be monitored as an index of the chronic inflammatory index. IL-1β, IL-8, and TNF-α were selected as the indices for non-specific inflammatory responses. IL-1β is an important mediator of the inflammatory response. When IL-1β is secreted, it circulates systemically and is involved in non-specific inflammation [30]. IL-8 is a typical cytokine, which continuously stimulates multiple inflammatory signals [80]. Recently, IL-8 has been studied extensively in relation to allergic dermatitis, including psoriasis. Also, TNF-α is a typical inflammatory cytokine produced in macrophages, T/B cells, NK cells, neutrophils, mast cells, endothelial cells, adipocytes, and epidermal keratinocytes [81]. When TNF-α is secreted, it is involved in various immune responses, including cell proliferation, differentiation, and apoptosis [82]. TNF-α, the levels of which are elevated in the serum and skin, especially in patients with AD, is thought to play an important role in the regulation of inflammation in AD and psoriasis [83,84]. The treatment of inflammatory stimulants to HDFs and RAW264.7 cells can be used as a model to induce non-specific inflammatory responses. Therefore, this was a suitable method to observe how AS extracts acted on non-specific inflammation reactions. IL-33 is known to act as an inflammatory alarmin/early inflammatory regulator [85] and is an important cytokine in Th2-mediated immune responses that plays a central role in the regulation of immune responses in barrier tissues, such as the skin and the bowel [86]. It can activate many immune cells involved in the induction of acute AD and the conversion of acute to chronic AD [87]. It is an excellent index on which to judge the disease progression and development of inflammation and chronic AD.

In RAW264.7 cells, IL-10 was upregulated by all inflammatory stimulants (LPS, TNF-α, and IFN-γ); TNF-α increased IL-1β; and IFN-γ increased IL-6. These results implied that macrophages were involved in both the acute and non-specific inflammation induced by exposure to inflammatory stimulants [88,89]. Sato et al. [90] reported that the stimulation of macrophages with LPS increased IL-33 expression. The same result was obtained in our study. It is known that an increase in IL-33 leads to an increase in the production of inflammatory cytokines (especially IL-10), leading to massive induction of inflammation [28,32]. The inhibition of IL-33 expression may be a good target for the suppression of the inflammatory response. The results of this study showed that macrophages are involved in all the processes, from non-specific inflammation to acute inflammation, the detection of the onset of inflammation, and the spread of massive inflammation. Therefore, macrophages are a good model in which to observe the effects of anti-inflammatory substances. In this study, we also examined the effects of AS extracts in macrophages.

Fibroblasts can mediate the immune response through the secretion of cytokines, chemokines, and growth factors. We observed that acute inflammatory cytokines (IL-6), alarmin cytokines (IL-33), and non-specific cytokines (IL-1β and IL-8) were upregulated by treatment with inflammatory stimulants in HDFs. A result of particular interest was the increased expression of IL-33 induced by IFN-γ. Prefontaine et al. [4] and Meephansan et al. [91,92] reported that IL-33 expression was increased by IFN-γ alone or by the combination treatment of TNF-α and IFN-γ in airway epithelial cells and normal epithelial Kc (NEK) cells, respectively. According to Seltmann et al. [93], the simultaneous treatment of TNF-α and IFN-γ in Kc cells increased the expression of IL-33; this increased expression of IL-33 forms a positive feedback loop that, in turn, promotes IFN-γ secretion. This loop acts as an important mechanism for the conversion from acute to chronic inflammation. TNF-α is also considered a cytokine that is induced by IL-33 and exacerbates inflammation. In this study, the expression of IL-33 was increased not only by IFN-γ but also by TNF-α and LPS treatment, suggesting that the positive loop in Kc, as claimed by Seltmann et al. was also present in HDFs. In RAW264.7 cells, IL-33 was increased only after stimulation with LPS. As a result, HDFs, which are typically considered as simple tissue repair cells, may have a greater role as immune cells than macrophages and Kc cells during the conversion from acute to chronic inflammation.

## 4. Materials and Methods

### 4.1. Plant Material, Extraction, and Isolation

The barks and leaves of AS were purchased from KGC Yebon (Chungju, Korea), and were collected from Songrim, Hamyang, and Jacheon, Korea. The voucher specimens were deposited at the herbarium of the College of Pharmacy, Chung-Ang University (Seoul, Korea). Two hundred grams each of the dried barks and leaves of AS from Songrim, Hamyang, and Jacheon were extracted with 70% ethanol A at room temperature (25 °C) three times for 3 days to yield the SB, HB, and JB, and SL, HL, and JL extracts, respectively (Table 1). The barks and leaves from Jacheon (450 g each) were extracted with 70% proethanol A (Cat. No. J5V108, Reagents DUKSAN, Gyeonggi, Korea) at 70 °C and refluxed three times to yield the JBR and JLR extracts (Table 1). JBR extract (50 g) and JLR extract (22 g) were fractionated by using ethyl acetate (EA) to yield JBRE and JLRE fractions (Table 1). The extract yields after the removal of proethanol A and EA under vacuum were: SB, 17.76 g; HL, 36.03 g; JL, 21.55 g; HB, 17.22 g; JB, 14.93 g; JBR, 48.28 g; JLR, 101 g; JBRE, 5.35 g; and JLRE, 20.22 g. AS extracts were dissolved in 70% and 100% ethanol to produce stock solutions of 100,000 and 500,000 μg/mL and diluted to the concentration required for use in experiments.

### 4.2. HPLC Analysis

High-performance liquid chromatography (HPLC) (Waters 600 system (Marchall Scientific, Hampton, NH, USA) and a Hector C^18^ (5 μm, 4.6 × 250 mm) Column were used for quantitative surveys of the contents of the compounds. The mobile phase consists of solvents A (H_2_O) and B (Acetonitrile, ACN), which were described and filtered by Whatman^®^ membrane filters (0.2 μm, diam. 47 mm) (Table 2).

### 4.3. Measurement of DPPH Radical Scavenging Activity

Each 20 μL sample was added to 0.2 mM of DPPH (180 μL; Sigma-Aldrich, St. Louis, MO, USA) in absolute ethanol. The samples were mixed gently and left to stand for 30 min. The optical density (OD) of the samples at 518 nm was measured using an ELISA reader (Tecan Sunrise, Salzburg, Austria). The free radical scavenging activity was calculated as the inhibition rate (%) = [1 − (sample O.D./control O.D.)] × 100; ascorbic acid was used as the positive control.

### 4.4. Cell Culture

Primary HDFs (Cat. C-013-5C, GIBCO Cascade Biologics^®^, Waltham, MA, USA) were cultured in Medium 106 (M106, Cat. M106-500) containing Low Serum Growth Supplement Kits (LSGS kit, Cat. S-003-K). Medium and supplement kits were purchased from Invitrogen Life Technologies Inc. (GIBCO Cascade Biologics^®^, Waltham, MA, USA). RAW264.7 cells (KCLB40071, Lot. No. 27186, Seoul, Korea) were purchased from the Korean Cell Line Bank. RAW264.7 cells were cultured in Dulbecco’s modified Eagle’s medium (DMEM) containing 10% fetal bovine serum (FBS) and 100 IU/mL penicillin-streptomycin. Medium and antibiotics were purchased from Welgene (Daegu, Korea). The cells were maintained in culture flasks at 37 °C in a humidified atmosphere with 5% CO_2_. For all the experiments, the cells were grown to greater than 80% confluence and subjected to no more than 15 passages.

### 4.5. Measurement of Cell Viability

Approximately 1 × 10^5^ cells/well HDFs or RAW264.7 cells were seeded in each well of a 96-well plate and incubated overnight in an atmosphere of 5% CO_2_ at 37 °C. Subsequently, the medium was replaced with phosphate-buffered saline (PBS) containing 5 mg/mL of MTT and incubated for 3 h. The supernatant was removed, and 100 μL of dimethyl-sulfoxide (DMSO) was added to solubilize the formed formazan crystals. We measured the reduction of MTT to formazan within the cells through at 540 nm by using a microplate reader (Sunrise; TECAN, Salzburg, Austria). The cell viability was calculated as the sample OD/blank OD × 100 (%).

### 4.6. Inflammatory Stimulants and AS Extract Treatments

HDFs and RAW264.7 cells were seeded at a density of 5 × 10^5^ cells per plate in 10 mL in a 10-mm culture dish and incubated at 37 °C in 5% CO_2_ overnight to adhere. The medium was removed, and the cells were treated with 300 μg/mL or 1000 μg/mL AS extracts (No. 2, 4, 8, 9) or medium for 1 h before treatment with inflammatory stimulants. The inflammatory stimulants added to the cells were 100 ng/mL of LPS for 3 h, 10 ng/mL of TNF-α for 12 h, or 10 ng/mL of IFN-γ for 12 h.

### 4.7. RNA Isolation and cDNA Synthesis

Total RNA was isolated from the cells by using TRIzol reagent (Invitrogen, Waltham, MA, USA) and left at room temperature for 5 min. Subsequently, 0.2 mL of chloroform (Sigma-Aldrich, St. Louis, MO, USA) was added, and the mixture was shaken vigorously for 15 s and then centrifuged at 12,000× *g* for 15 min at 4 °C. The supernatants were transferred to a new tube with 70% ethanol. RNA purification using the PureLink^®^ RNA Mini Kit (Ambion^®^, Carlsbad, California, CA, USA) was performed after the isolation of total RNA. The yield and quality of total RNA were analyzed by using a Nanodrop spectrophotometer (Thermo Scientific, Waltham, MA, USA). Purified total RNA (2 µg) was added to the Eco Dry^TM^ premix (oligo dT) kit (TaKaRa, Shiga, Japan), and the total reaction volume was adjusted to 20 μL to synthesize cDNA by PCR. PCR was conducted by using S1000 Thermal Cycler (Bio-Rad, Hercules, CA, USA) in accordance with the manufacturer’s instructions.

### 4.8. Reverse-Transcriptase Polymerase Chain Reaction

We designed the primer sequences based on known mRNA sequences (Table 3) by using Entrez (The National Center for Biotechnology Information, NCBI; The National Institutes of Health, NIH) and performed reverse transcription using the TaKaRa Ex Taq kit (Takara, Shiga, Japan) in accordance with the manufacturer’s instructions. The cDNA was amplified for 30 cycles of reverse transcription by using an S1000 Thermal Cycler (Bio-Rad, Hercules, CA, USA). The amplification steps were pre-heating (5 min at 95 °C), 35 cycles of 60 s at 95 °C, 60 s at each primer’s annealing temperature, 60 s at 72 °C, and a final extension (72 °C for 5 min). The PCR reaction products were resolved on a 1.2% agarose gel, stained with ethidium bromide (EtBr), and visualized under ultraviolet (UV) light. Various cytokines (IL-1β, IL-6, IL-18, IL-33, and TNF-α) were observed, in addition to IL-8 in HDFs and IL-10 in RAW264.7 cells.

### 4.9. Statistical Analysis

All the data are expressed as the mean ± standard deviation (SD). All the analyses were performed on six independent experiments, and each result was analyzed in three replicates. Statistical significance was assessed by *t*-tests.

## 5. Conclusions

Skin inflammation is characterized by the production of cytokines/chemokines and the skin infiltration of immune cells. Changes in their expression are important, because cytokines and chemokines play essential roles in inflammation and inflammatory cell activities [80,94,95]. Although this area is well studied, the interpretation of skin inflammation is still unclear. The results of this study suggest that the treatment of inflammatory stimulants in HDFs and RAW264.7 cells and the subsequent changes in the expression of cytokines can be used as an excellent in vitro model of inflammatory induction to confirm non-specific, acute, and chronic inflammatory responses, and the acute to chronic inflammatory conversion. In this study, we investigated the effects of AS extracts on the expression of cytokines by using this model. The increased expression of most cytokines induced by inflammatory stimulants and the physiological expression of most cytokines in HDFs and RAW264.7 cells were inhibited by all the AS extracts. IL-33, which links acute and chronic inflammation, was also inhibited by all the AS extracts. Therefore, it is suggested that these AS extracts may be excellent therapeutic agents for the suppression of the acute phase of skin inflammation and the inhibition of the transition from acute to chronic inflammation, thereby suppressing overall skin inflammation.

## Figures and Tables

**Table 1:** Information on *Alnus sibirica* (AS) extracts used in experiments.

No.	Name	Substrate
AS1	SB	Barks, Songrim, Korea
AS2	HL	Leaves, Hamyang, Korea
AS3	JL	Leaves, Jacheon, Korea
AS4	HB	Barks, Hamyang, Korea
AS5	JB	Barks, Jacheon, Korea
AS6	JBR	Barks, reflux extraction, Jacheon, Korea
AS7	JLR	Leaves reflux extraction, Jacheon, Korea
AS8	JBRE	Barks, reflux extraction, and EA fraction, Jacheon, Korea
AS9	JLRE	Leaves, reflux extraction, and EA fraction, Jacheon, Korea

**Table 2:** Analysis condition of HPLC.

Column	Hector C^18^ HPLC Column (5 µm, 250 × 4.6 mm)						
Detector	Waters 112 UV/V_IS_ (280 nm)						
	0 min	20 min	35 min	38 min	45 min	47 min	55 min
H_2_O	95	75	60	0	0	95	95
ACN	5	25	40	100	100	5	5

**Table 3:** Primer sequences. bp, base pair of product sizes; °C, annealing temperature; h, human; m, mouse; GAPDH, glyceraldehyde 3-phosphate dehydrogenase.

Primer	bp	°C	Sense	Antisense
hGAPDH	343	58	5′-CAGTGAGCTTCCCGTTCAG-3′	5′-GCCAAAAGGGTCATCATCTC-3′
hIL-1β	391	62	5′-AAACAGATGAAGTGCTCCTTCCAGG-3	5′-TGGAGAACACCACTTGTTGCTCCA-3′
hIL-6	264	62	5′-ATGAACTCCTTCTCCACAAGC-3′	5′-GTTTTCTGCCAGTGCCTCTTTG-3′
hIL-8	293	58	5′-ATGACTTCCAAGCTGGCCGTGGCT-3′	5′-TCTCAGCCCTCTTCAAAAACTTCTC-3′
hIL-18	342	53	5′-GCTTGAATCTAAATTATCAGTC-3′	5′-GAAGATTCAAATTGCATCTTAT-3′
hIL-33	180	58	5′-CAAAGAAGTTTGCCCCATGT-3′	5′-AAGGCAAAGCACTCCACAGT-3′
hTNF-α	237	62	5′-GAGCTGAGAGATAACCAGCTGGTG-3′	5′-CAGATAGATGGGCTCATACCAGGG-3′
mGAPDH	343	58	5′-CAGTGAGCTTCCCGTTCAG-3′	5′-GCCAAAAGGGTCATCATCTC-3′
mIL-1β	213	53	5′-AAGGAGAACCAAGCAACGACAAAA-3′	5′-TGGGGAACTCTGCAGACTCAAACT-3′
mIL-6	141	62	5′-AGGATACCACTCCCAACAGACCT-3′	5′-CAAGTGCATCATCGTTGTTCATAC-3′
mIL-10	252	55	5′-TACCTGGTAGAAGTGATGCC-3′	5′-CATCATGTATGCTTCTATGC-3′
mIL-18	434	53	5′-ACTGTACAACCGCAGTAATACGC-3′	5′-AGTGAACATTACAGATTTATCCC-3′
mIL-33	155	61	5′-GGTGTGGATGGGAAGAAGCTG-3′	5′-GAGGACTTTTTGTGAAGGACG-3′
mTNF-α	384	61	5′-GCGACGTGGAACTGGCAGAAG-3′	5′-GGTACAACCCATCGGCTGGCA-3′

## References

[1] Kessler-Becker D., Krieg T., Eckes B. (2004). Expression of pro-inflammatory markers bu human dermal fibroblasts in a three-dimensional culture model is mediated by an autocrine interleukin-1. Biochem. J..

[2] Pasparakis M., Haase I., Nestle F.O. (2014). Mechanisms regulating skin immunity and inflammation. Nat. Rev. Immunol..

[3] Schulte W., Bernhagen J., Bucala R. (2013). Cytokines in sepsis: Potent immunoregulators and potential therapeutic target—An updated view. Mediat. Inflamm..

[4] Prefontaine D., Lajoie-Kadoch S., Foley S., Audusseau S., Olivenstein R., Halayko A.J., Lemiere C., Martin J.G., Hamid Q. (2009). Increased expression of IL-33 in severe asthma: Evidence of expression by Airway smooth muscle cells. J. Immunol..

[5] Chen L., Deng H., Cui H., Fang J., Zuo Z., Deng J., Li Y., Wang X., Zhao L. (2018). Inflammatory responses and inflammation-associated diseases in organs. Oncotarget.

[6] Sugimoto M.A., Sousa L.P., Pinho V., Perretti M., Teixeira M.M. (2016). Resolution of inflammation: What controls its onset?. Front. Immunol..

[7] Malemud C.J. (2018). The role of the JAK/STAT signal pathway in rheumatoid arthritis. Ther. Adv. Musculoskelet. Dis..

[8] Packard R.R.S., Libby P. (2008). Inflammation in atherosclerosis: From vascular biology to biomarker discovery and risk prediction. Clin. Chem..

[9] Wittmann M., McGonagle D., Werfel T. (2014). Cytokines as therapeutic targets in skin inflammation. Cytokine Growth Factor Rev..

[10] Nedorost S.T. (2012). Generalized Dermatitis in Clinical Practice.

[11] Roeleveld D.M., Koenders M.I. (2015). The role of the Th17 cytokines IL-17 and IL-22 in Rheumatoid Arthritis pathogenesis and developments in cytokine immunotherapy. Cytokine.

[12] Zhang H.-L., Zheng X.-Y., Zhu J. (2013). Th1/Th2/Th17/Treg cytokines in Guillain–Barré syndrome and experimental autoimmune neuritis. Cytokine Growth Factor Rev..

[13] Ayakannu R., Abdullah N., Radhakrishnan A.K., Raj V.L., Liam C. (2019). Relationship between various cytokines implicated in asthma. Hum. Immunol..

[14] Howard M., Muchamuel T., Andrade S., Menon S. (1993). Interleukin 10 protects mice from lethal endotoxemia. J. Exp. Med..

[15] Nicoletti F., Mancuso G., Cusumano V., Marco R.D., Zaccone P., Bendtzen K., Teti G. (1997). Prevention of endotoxin-induced lethality in neonatal mice by interleukin-13. Eur. J. Immunol..

[16] Gómez D., Correa P.A., Gómez L.M., Cadena J., Molina J.F., Anaya J.-M. (2004). Th1/Th2 cytokines in patients with systemic lupus erythematosus: Is tumor necrosis factor α protective?. Semin. Arthritis Rheum..

[17] Chakraborty S., Kubatzky K.F., Mitra D.K. (2019). An Update on Interleukin-9: From Its Cellular Source and Signal Transduction to Its Role in Immunopathogenesis. Int. J. Mol. Sci..

[18] Deng Y., Wang Z., Chang C., Lu L., Lau C.S., Lu Q. (2017). Th9 cells and IL-9 in autoimmune disorders: Pathogenesis and therapeutic potentials. Human Immunol..

[19] Nicoletti F., Di Marco R., Patti F., Reggio E., Nicoletti A., Zaccone P., Stivala F., Meroni P., Reggio A. (1998). Blood levels of transforming growth factor-beta 1 (TGF-β1) are elevated in both relapsing remitting and chronic progressive multiple sclerosis (MS) patients and are further augmented by treatment with interferon-beta 1b (IFN-β1b). Clin. Exp. Immunol..

[20] Li M.O., Wan Y.Y., Sanjabi S., Robertson A.-K.L., Flavell R.A. (2006). Transforming growth factor-β regulation of immune responses. Annu. Rev. Immunol..

[21] Leung D.Y.M., Bieber T. (2003). Atopic dermatitis. Lancet.

[22] Wollenberg A., Bieber T. (2000). Atopic dermatitis from the genes to skin lesions. Allergy.

[23] Dokmeci E., Herrick C.A. (2008). The immune system and atopic dermatitis. Semin. Cutan. Med. Surg..

[24] National Institute of Arthritis and Musculoskeetal and Skin Diseases (1999). Handout on Health.

[25] Lee A.Y. (2010). Immunologic barrier in skin of atopic dermatitis. Atopic Dermat. Symp..

[26] Kalluri R., Zeisberg M. (2006). Fibroblasts in cancer. Nat. Rev. Cancer.

[27] Van Linthout S., Miteva K., Tschope C. (2014). Crosstalk between fibroblasts and inflammatory cells. Cardiovasc. Res..

[28] Murray P.J., Wynn T.A. (2011). Protective and pathogenic functions of macrophage subsets. Nat. Rev. Immunol..

[29] Taniguchi K., Yamamoto S., Hitomi E., Inada Y., Suyama Y., Sugioka T., Hamasaki Y. (2013). Interleukin 33 is induced by tumor necrosis factor A and interferon G in keratinocytes and contributes to allergic contact dermatitis. J. Investig. Allergol. Clin. Immunol..

[30] Sims J.E., Smith D.E. (2010). The IL-1 family: Regulators of immunity. Nat. Rev. Immunol..

[31] Moritz D.R., Gheyselinck J., Klemenz R., Rodewald H.R. (1998). The IL-1 receptor-related T1 antigen is expressed on immature and mature mast cells and on fetal blood mast cell progenitors. J. Immunol..

[32] Xie Q., Shen W.-W., Zhong J., Huang C., Zhang L., Li J. (2014). Lipopolysaccharide/adenosine triphosphate induces IL-1 beta and IL-18 secretion through the NLRP3 inflammasome in RAW264.7 murine macrophage cells. Int. J. Mol. Med..

[33] Santarlasci V., Cosmi L., Maggi L., Liotta F., Annunziato F. (2013). IL-1 and T helper immune responses. Front. Immunol..

[34] Dinarello C.A. (2009). Immunological and inflammatory functions of the interleukin-1 family. Ann. Rev. Immunol..

[35] Delgado A.V., McManus A.T., Chambers J.P. (2003). Production of tumor necrosis factor-alpha, interleukin 1-beta, interleukin 2, and interleukin 6 by rat leukocyte subpopulations after exposure to Substance P. Neuropeptides.

[36] Matsushima K., Morishita K., Yoshimura T., Lavu S., Kobayashi Y., Lew W., Appella E., Kung H.F., Leonard E.J., Oppenheim J.J. (1988). Molecular cloning of a human monocyte-derived neutrophil chemotactic factor (MDNCF) and the induction of MDNCF mRNA by interleukin 1 and tumor necrosis factor. J. Exp. Med..

[37] Baggiolini M., Walz A., Kunkel S.L. (1989). Neutrophil-activating peptide-1/interleukin 8, a novel cytokine that activates neutrophils. J. Clin. Investig..

[38] Strieter R.M., Koch A.E., Antony V.B., Fick R.B., Standiford T.J., Kunkel S.L. (1994). The immunopathology of chemotactic cytokines: The role of interleukin-8 and monocyte chemoattractant protein-1. J. Lab. Clin. Med..

[39] Dembic Z. (2015). The Cytokines of the Immune System: The Role of Cytokines in Disease Related to Immune Response.

[40] Barbulescu K., Becker C., Schlaak J.F., Schmitt E., Meyer zum Büschenfelde K.H., Neurath M.F. (1998). IL-12 and IL-18 differentially regulate the transcriptional activity of the human IFN-gamma promoter in primary CD4+ T lymphocytes. J. Immunol..

[41] Coondoo A. (2011). Cytokines in dermatology—A basic overview. Indian J. Dermatol..

[42] Löhning M., Stroehmann A., Grogan J.L., Radbruch A., Kamradt T., Coyle A.J., Lin S., Gutierrez-Ramos J.C., Levinson D. (1998). T1/ST2 is preferentially expressed on murine Th2 cells, independent of interleukin 4, interleukin 5, and interleukin 10, and important for Th2 effector function. Proc. Natl. Acad. Sci. USA.

[43] Stolarski B., Kurowska-Stolarska M., Kewin P., Xu D., Liew F.Y. (2010). IL-33 exacerbates eosinophil-mediated airway inflammation. J. Immunol..

[44] Schneider E., Petit-Bertron A.-F., Bricard R., Levasseur M., Ramadan A., Girard J.-P., Herbelin A., Dy M. (2009). IL-33 activates unprimed murine basophils directly in vitro and induces their in vivo expansion indirectly by promoting hematopoietic growth factor production. J. Immunol..

[45] Bourgeois E., Van L.P., Samson M., Diem S., Barra A., Roga S., Gombert J.-M., Schneider E., Dy M., Gourdy P. (2009). The pro-Th2 cytokine IL-33 directly interacts with invariant NKT and NK cells to induce IFN-gamma production. Eur. J. Immunol..

[46] Rank M.A., Kobayashi T., Kozaki H., Bartemes K.R., Squillace D.L., Kita H. (2009). IL-33–activated dendritic cells induce an atypical T H2-type response. J. Allerg. Clin. Immunol..

[47] Saluja R., Khan M., Church M.K., Maurer M. (2015). The role of IL-33 and mast cells in allergy and inflammation. Clin. Transl. Allerg..

[48] Weidinger S., Beck L.A., Bieber T., Kabashima K., Irvine A.D. (2018). Atopic dermatitis. Nat. Rev. Dis. Prim..

[49] Elias M.S., Long H.A., Wu K.C., Reynolds N.J., Newman C.F., Wilson P.A., West A., McGill P.J., Donaldson M.J. (2017). Proteomic analysis of filaggrin deficiency identifies molecular signatures characteristic of atopic eczema. J. Allerg. Clin. Immunol..

[50] Tracey M.D.K.J., Cerami P.D.A. (1994). Tumor necrosis factor: A pleiotropic cytokine and therapuetic target. Ann. Rev. Med..

[51] Luedde T., Schwabe R.F. (2011). NF-kappaB in the liver--linking injury, fibrosis and hepatocellular carcinoma. Nat. Rev. Gastroenterol. Hepatol..

[52] Lee S.-J. (1966). Korean folk medicine. Korean J. Pharmacogn..

[53] Pui-hay B.P., Sung C.K. (2001). International Collation of Traditional and Folk Medicine.

[54] Kim M.H., Park K.H., Kim S.R., Park K.J., Oh M.H., Heo J.H., Yoon K.H., Yin J., Yoon K.H., Lee M.W. (2016). Two new phenolic compounds from the leaves of *Alnus sibirica* Fisch. ex Turcz. Nat. Prod. Res..

[55] Suga T., Iwata N., Asakawa Y. (1972). Chemical constituents of the male flower of *Alnus pendula* (BETULACEAE). Bull. Chem. Soc. Jpn..

[56] Choi S.E., Kim K.H., Kwon J.H., Kim S.B., Kim H.W., Lee M.W. (2008). Cytotoxic activities of diarylheptanoids from *Alnus japonica*. Arch. Pharm. Res..

[57] Choi E.J., Ko H.H., Lee M.W., Bang H., Lee C.S. (2008). Inhibition of activated responses in dendritic cells exposed to lipopolysaccharide and lipoteichoic acid by diarylheptanoid oregonin. Int. Immunopharmacol..

[58] Lee M., Kim N., Park M., Ahn K., Toh S., Hahn D., Kim Y., Chung H. (2000). Diarylheptanoids with in vitro inducible nitric oxide synthesis inhibitory activity from *Alnus hirsuta*. Planta Med..

[59] Choi S.E., Park K.H., Jeong M.S., Kim H.H., Lee D.I., Joo S.S., Lee C.S., Bang H., Choi Y.W., Lee M.K. (2011). Effect of *Alnus japonica* extract on a model of atopic dermatitis in NC/Nga mice. J. Ethnopharmacol..

[60] Suga T., Ohta S., Ohta E., Aoki T. (1981). A C31-secodammarane-type triterpenic acid, 12-deoxy alnustic acid, from the female flowers of *alnus pendula*. Phytochemistry.

[61] Nomura M., Tokoroyama T., Kubota T. (1981). Biarylheptanoids and other constituents from wood of *Alnus japonica*. Phytochemistry.

[62] Lee M., Pak M., Jeong D., Kim K., Kim H., Toh S. (2000). Diarylheptanoids from the leaves of *Alnus hirsuta* Turcz. Arch. Pharm. Res..

[63] Joo S.S., Kim S.G., Choi S.E., Kim Y.B., Park H.Y., Seo S.J., Choi Y.W., Lee M.W., Lee D.I. (2009). Suppression of T cell activation by hirsutenone, isolated from the bark of *Alnus japonica*, and its therapeutic advantages for atopic dermatitis. Eur. J. Pharmacol..

[64] Joo S.-S., Kim M.-S., Oh W.-S., Lee D.-I. (2002). Enhancement of NK cytotoxicity, antimetastasis and elongation effect of survival time in B16-F10 melanoma cells by oregonin. Arch. Pharm. Res..

[65] Yin J., Yoon S.H., Ahn H.S., Lee M.W. (2018). Inhibitory activity of allergic contact dermatitis and atopic dermatitis-like skin in BALB/c mouse through oral administration of fermented barks of *Alnus sibirica*. Molecules.

[66] Leung D.Y. (2000). Atopic dermatitis: New insights and opportunities for therapeutic intervention. J. Allerg. Clin. Immunol..

[67] Narazaki M., Kishimoto T. (2018). The two-faced cytokine IL-6 in host defense and diseases. Int. J. Mol. Sci..

[68] Kishimoto T. (2010). IL-6: From its discovery to clinical applications. Int. Immunol..

[69] Hsu D.H., Moore K.W., Spits H. (1992). Differential effects of IL-4 and IL-10 on IL-2-induced IFN-gamma synthesis and lymphokine-activated killer activity. Int. Immunol..

[70] Chung E.Y., Liu J., Homma Y., Zhang Y., Brendolan A., Saggese M., Han J., Silverstein R., Selleri L., Ma X. (2007). Interleukin-10 expression in macrophages during Phagocytosis of apoptotic cells is mediated by homeodomain proteins Pbx1 and Prep-1. Immunity.

[71] Fiorentino D.F., Bond M.W., Mosmann T.R. (1989). Two types of mouse t helper cell. IV. Th2 clones secrete a factor that inhibits cytokine production by Thl clones. J. Exp. Med..

[72] Moore K.W., Vieira P., Fiorentino D.F., Trounstine M.L., Khan T.A., Mosmann T.R. (1990). Homology of cytokine synthesis inhibitory factor (IL-10) to the Epstein-Barr virus gene BCRFI. Science.

[73] Fuss I.J., Boirivant M., Lacy B., Strober W. (2002). The interrelated roles of TGF-beta and IL-10 in the regulation of experimental colitis. J. Immunol..

[74] Kühn R., Löhler J., Rennick D., Rajewsky K., Müller W. (1993). Interleukin-10-deficient mice develop chronic enterocolitis. Cell.

[75] Berg D.J., Leach M.W., Kühn R., Rajewsky K., Müller W., Davidson N.J., Rennick D. (1995). Interleukin 10 but not interleukin 4 is a natural suppressant of cutaneous inflammatory responses. J. Exp. Med..

[76] Berg D.J., Davidson N., Grünig G., Rennick D., Kühn R., Rajewsky K., Müller W., Menon S. (1995). Interleukin-10 is a central regulator of the response to LPS in murine models of endotoxic shock and the Shwartzman reaction but not endotoxin tolerance. J. Clin. Investig..

[77] Bettelli E., Das M.P., Howard E.D., Weiner H.L., Kuchroo V.K., Sobel R.A. (1998). IL-10 is critical in the regulation of autoimmune encephalomyelitis as demonstrated by studies of IL-10- and IL-4-deficient and transgenic mice. J. Immunol..

[78] Wittmann M., Macdonald A., Renne J. (2009). IL-18 and skin inflammation. Autoimmun. Rev..

[79] Gracie J.A., Robertson S.E., McInnes I.B. (2003). Interleukin-18. J. Leukoc. Biol..

[80] Nedoszytko B., Sokołowska-Wojdyło M., Roszkiewicz J., Nowicki R.J., Ruckemann-Dziurdzińska K. (2014). Chemokines and cytokines network in the pathogenesis of the inflammatory skin diseases: Atopic dermatitis, psoriasis and skin mastocytosis. Postep. Dermatol. Alergol..

[81] Zelova H., Hosek J. (2013). TNF-alpha signalling and inflammation: Interactions between old acquaintances. Inflamm. Res..

[82] Monaco C., Nanchahal J., Taylor P., Feldmann M. (2015). Anti-TNF therapy: Past, present and future. Int. Immunol..

[83] Mizuno K., Morizane S., Takiguchi T., Iwatsuki K. (2015). Dexamethasone but not tacrolimus suppresses TNF-alpha-induced thymic stromal lymphopoietin expression in lesional keratinocytes of atopic dermatitis model. J. Dermatol. Sci..

[84] Junghans V., Gutgesell C., Jung T., Neumann C. (1998). Epidermal cytokines IL-1β, TNF-α, and IL-12 in patients with atopic dermatitis: Response to application of house dust mite antigens. J. Investig. Dermatol..

[85] Cayrol C., Girard J.P. (2014). IL-33: An alarmin cytokine with crucial roles in innate immunity, inflammation and allergy. Curr. Opin. Immunol..

[86] Miller A.M. (2011). Role of IL-33 in inflammation and disease. J. Inflamm..

[87] Nabe T. (2014). Interleukin (IL)-33: New therapeutic target for atopic diseases. J. Pharmacol. Sci..

[88] Wynn T.A., Vannella K.M. (2016). Macrophages in tissue repair, regeneration, and fibrosis. Immunity.

[89] Hung Y.-L., Fang S.-H., Wang S.-C., Cheng W.-C., Liu P.-L., Su C.-C., Chen C.-S., Huang M.-Y., Hua K.-F., Shen K.-H. (2017). Corylin protects LPS-induced sepsis and attenuates LPS-induced inflammatory response. Sci. Rep..

[90] Sato S., Yanagawa Y., Hiraide S., Iizuka K. (2016). Cyclic AMP signaling enhances lipopolysaccharide sensitivity and interleukin-33 production in RAW264.7 macrophages. Microbiol. Immunol..

[91] Meephansan J., Tsuda H., Komine M., Tominaga S., Ohtsuki M. (2012). Regulation of IL-33 expression by IFN-gamma and tumor necrosis factor-alpha in normal human epidermal keratinocytes. J. Investig. Dermatol..

[92] Meephansan J., Komine M., Tsuda H., Karakawa M., Tominaga S.-I., Ohtsuki M. (2013). Expression of IL-33 in the epidermis: The mechanism of induction by IL-17. J. Dermatol. Sci..

[93] Seltmann J., Werfel T., Wittmann M. (2013). Evidence for a regulatory loop between IFN-γ and IL-33 in skin inflammation. Exp. Dermatol..

[94] Shaik-Dasthagirisaheb Y.B., Conti P. (2015). Chemokine network involved in inflammatory skin diseases. Ann. Clin. Lab. Sci..

[95] Borish L.C., Steinke J.W. (2003). Cytokines and chemokines. J. Allerg. Clin. Immunol..

